# Associations between dietary acid load and obesity among Iranian women

**DOI:** 10.34172/jcvtr.2021.44

**Published:** 2021-08-28

**Authors:** Somaye Fatahi, Mostafa Qorbani, Pamela J. Surkan, Leila Azadbakht

**Affiliations:** ^1^Department of Community Nutrition, School of Nutritional Sciences and Dietetics, Tehran University of Medical Sciences, Tehran, Iran; ^2^Pediatric Gastroenterology, Hepatology and Nutrition Research Center, Research Institute for Children’s Health, Shahid Beheshti University of Medical sciences, Tehran, Iran; ^3^Non-Communicable Diseases Research Center, Alborz University of Medical Sciences, Karaj, Iran; ^4^Department of International Health John Hopkins Bloomberg School of Public Health, Baltimore, USA; ^5^Diabetes Research Center, Endocrinology and Metabolism Clinical Sciences Institute, Tehran University of Medical Sciences, Tehran, Iran

**Keywords:** Dietary Acid Load, Overweight, Obesity, Diet, Nutrition

## Abstract

***Introduction:*** Diet-induced acid load may be associated with overweight and obesity as well as with diet quality. We aimed to study how dietary acid load is associated with overweight, obesity and diet quality indices in healthy women.

***Methods:*** We randomly selected 306 healthy 20 to 55 year-old women from health centers affiliated with Tehran University of Medical Science. They were enrolled in a cross-sectional study between June2016 and March 2017. Potential renal acid load (PRAL), net endogenous acid production (NEAP) and dietary acid load (DAL) were calculated for each person. Dietary quality index international (DQI-I),mean adequacy ratio (MAR), and energy density (ED) were estimated. Anthropometry was measured using standard protocols. Nutritional data were obtained from food frequency questionnaires (FFQ). We used multivariable logistic regression models to assess dietary acid load indices in relation to overweight, obesity and abdominal adiposity.

***Results:*** Participants had a mean age of 32.4 years. The number and percentage of women who were overweight, obese and who had abdominal obesity were 94(30.7), 38(12.4) and 126(41.2), respectively.The odds of obesity (adjusted odds ratio; Adj. OR = 2.41, 95% confidence interval; CI:1.01-5.74,*P* = 0.045) and abdominal adiposity (Adj. OR = 2.4, 95% CI:1.34-4.60, *P* = 0.004) increased significantly with tertile of DAL. Other dietary acid load indices (PRAL and NEAP) showed no significant association with obesity, overweight or abdominal obesity. As dietary acid load scores (PRAL, NEAP and DAL)increased, DQI-I and MAR significantly decreased whereas ED significantly increased across tertilesof dietary acid load indices (*P* < 0.001).

***Conclusion:*** Dietary acid load is associated with obesity and abdominal obesity and is also considered an indicator of diet quality.

## Introduction

Overweight and obesity are serious public health problems that contribute to chronic diseases worldwide, especially cardiovascular disease.^1-4^ The prevalence of these disorders has been increasing at an alarming rate across the globe.^5^ A recent meta-analysis documented that the prevalence of overweight and obesity were about 41% and 13% in Iranian adults, respectively.^6^ In another systematic review and meta-analysis, authors estimated that the prevalence of obesity in older adults was around 21% and that the proportion of overweight and obesity in women was higher than in men.^7^ Among the myriad genetics, environmental, and behavioral determinants associated with the rise of overweight and obesity, nutrition is a critical factor.^8,9^ Numerous studies have documented associations between the relative intake of different food groups (whole grains, dairy, fruits, and vegetables) and obesity.^10-12^ A person’s typical diet represents a mixture of food groups, in which foods and nutrients are consumed together. For that reason, an analysis of dietary patterns, rather than an analysis of food groups or individual foods, can contribute to our understanding of synergistic interactions between foods and nutrients.^13,14^

The Western diet has a strong influence on global eating patterns.^15^ In recent years, the possibility of changes in physiological acid-base balance due to the Western diet have attracted attention. Numerous studies show consumption of high-protein foods and insufficient fruit and vegetable intake can increase the body’s hydrogen ion load.^16-18^ These findings suggest that efforts to measure the net acid load from dietary intake may help refine our understanding of the effects of diet on human health.^19,20^

Potential renal acid load (PRAL), net endogenous acid production (NEAP) and dietary acid load (DAL) are used to estimate the acidogenic potential of foods and are used as indices to assess dietary acid load.^21-24^ A*cid*–base *imbalance*leads to metabolic changes accompanied by potential complications including insulin resistance, high cortisol levels, increased abdominal obesity and decreased insulin sensitivity.^3,25-27^ Recent studies have shown a positive correlation between high dietary acid load and increased risk of high blood pressure, cardiovascular disease, and diabetes mellitus.^21,28-30^ For example, a recent study by Han et al showed that individuals in the highest PRAL tertile were at increased risk of atherosclerotic cardiovascular disease (ASCVD) risk over a ten-year period and were at higher risk compared to those in the lowest PRAL tertile.^31^ Since obesity and overweight are also risk factors for heart disease, they should not be ignored.

Food quality indices such as the diet quality index - international (DQI-I), Energy Density (ED) and the Mean Adequacy Ratio (MAR) are instruments to evaluate adherence to dietary guidelines.^32-34^ The associations between some of these indices and obesity have also been investigated.^35,36^ Given the variety of dietary patterns in Iran, the aim of our study was to investigate dietary acid load in relation to overweight, obesity and dietary quality indices among Iranian women.

## Materials and Methods

**Figure 1:**
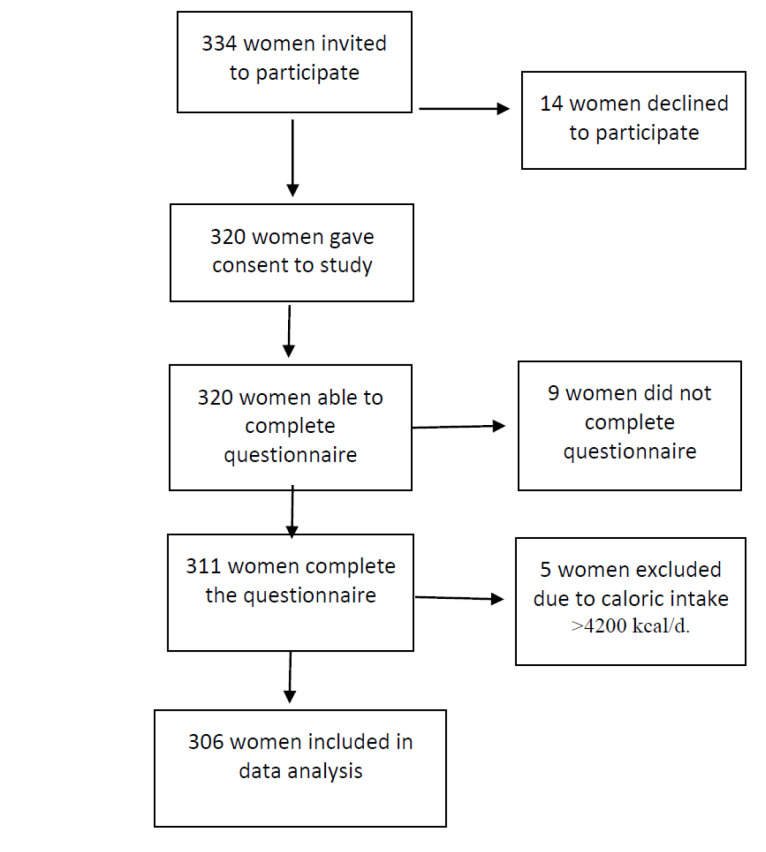
The flow diagram of study participants

The present study adhered to the Strengthening the Reporting of Observational Studies in Epidemiology (STROBE) guidelines for conducting and disseminating observational studies. Figure 1 displays a flow diagram of the study participants.

### Study design, setting and participants

We conducted a descriptive and analytical cross-sectional investigation with 20-55 year old women who were referred to health centers affiliated with Tehran University of Medical Science (TUMS) between June 2016 and March 2017. These women were selected by using systematic cluster random sampling. To this end, we divided the health centers into 15 clusters and systematically enrolled approximately 20 participants in each cluster. In total, 306 participants agreed to participate who had no prior history of cardiovascular disease (CVD), diabetes, cancer, stroke, chronic or acute renal disease, and who were not taking medications that could affect their weight. Exclusion criteria included being foreign (non-Iranian), pregnant or lactating. In addition, we excluded participants who reported total daily energy intake outside the 800-4200 kcal range. Information regarding demographic characteristics, health status, history of smoking, current medications and supplement intake was collected. Also, we obtained self-reported information on socio-economic status, which included ownership of nine household items (home, car, dishwasher, washing machine, LCD TV, refrigerator, woven carpet, laptop computer and microwave). Having < 3, 4 to 6, and > 7 of those household possessions were considered low, moderate and high socioeconomic status, respectively.^37^

### Sample size

We estimated sample size using the prevalence of overweight and obesity in adults who had the highest dietary acid load scores as the dependent variable^38^ employing the formula: N = [ [(z_1-α/2_) +(z_1-β_)]^2^ ×(p_1_(1-p_1_) +p_2_(1-p_2_)) ] / (p_2_-p_1_)^2^, where p_1_ = 36%, p_2_ = 52%, α = 0.05, β = 0.2 and where the ratio of normal weight to overweight/obese Iranian adults was 1.5.^39^ Based on this formula, we estimated that 306 participants were needed.

### Anthropometric indices

Body weight and height were measured using a tape measure and a standard scale with 100-gram accuracy. Anthropometry was assessed while participants wore light clothing and after removal of their shoes. Waist circumference (WC) was measured at the halfway point between the lower margin of the lowest rib and the top of the iliac crest and by using stretch‐resistant tape. Participants stood in a relaxed position with their feet close together and their arms at their sides. Body mass index (BMI) was calculated as weight (kg)/ height (m^2^). BMI ≥ 30 kg/ m^2^ and BMI = 25 to < 30 kg/m^2^, were defined as obesity and overweight respectively.^40^ Abdominal obesity was defined as WC > 88 cm.^41^

### Dietary intake

We collected dietary data using a 168-item food frequency questionnaire (FFQ) based on Willett et al.^42^ The reproducibility and validity of the FFQ has been evaluated specifically in Iranian adults.^43^ It includes a comprehensive list of diverse kinds of bread and grains, legumes, white and red meats, dairy products, fruits and vegetables, nuts, fats, and other foods. Food consumed per day, week, month, and year can be determined using this questionnaire.^44^ After administering the FFQ, we then converted the reported intakes to grams per day using standard published guidelines.^45^ Nutritionist IV software (Version 3.5.2) was used to estimate energy and macronutrient intake. A 24-hour physical activity record in MET was obtained from all women.^46^

### Dietary acid load

PRAL, NEAP and DAL scores were derived from nutrient intake equations. Tertiles of these scores were used for statistical analysis: PRAL (mEq/day) = (0.4888 × protein [g/day]) + (0.0366 × phosphorus [mg/day]) − (0.0205 × potassium [mg/day]) − (0.0263 × magnesium [mg/ day]) − (0.0125 × calcium [mg/day]),^22,47^ DAL (mEq/ day) = [(body surface area [m^2^] × 41 [mEq/ day]/ 1.73 m^2^ + PRAL].^48^ The Du Bois formula: [0.007184 × height^0.725^ × weight^0.425^] was used to calculate body surface area.^49,50^

### Diet quality

As another measure of dietary acid load, NEAP was calculated using following formula: NEAP(mEq/d) = [54:4 × protein intake (g/d)/potassium (mEq/d)] - 10.20].^51^ Using residual adjustment, we adjusted PRAL, NEAP and DAL for energy intake in the statistical analyses.

Nutrient adequacy was computed via the nutrient adequacy ratio (NAR).^52^ We calculated NAR for energy and for ten nutrients including vitamin C, vitamin A, vitamin B1, vitamin B2, phosphorus, iron, calcium, magnesium, zinc, and potassium. The mean probability of adequacy across these nutrients was obtained using Dietary Reference Intakes (DRIs). The NAR for nutrients is the ratio of a participant’s intake/current recommended allowance (RDA) for each age and sex category. Mean Adequacy Ratios (MAR) were also calculated with following formula:^53^


$$\mathrm{MAR}(\mathrm{Mean}\;\mathrm{Adequacy}\;\mathrm{Ratio})=\frac{\sum \mathrm{NAR}(\mathrm{selected}\;\mathrm{vitamns}\;\mathrm{and}\;\mathrm{minerals})}{\mathrm{Number}\;\mathrm{of}\;\mathrm{nutrients}}$$

Energy density (ED) was defined as the ratio of total daily energy intake (kcal/d) to total daily nutrient intake weight (g/d).^54^ The Diet Quality Index - International (DQI-I)^32^ was based on four aspects of a healthy diet, including (a) variety (overall and protein sources; 0-20 points), (b) adequacy (food/nutrient intake required to prevent undernutrition; 0-40 points), (c) moderation (food/nutrient intake quality relative to that associated with chronic disease; 0-30 points), and (d) balance (macronutrient ratio and fatty acid composition; 0-10 points). Variety was obtained from the sum of ‘a within protein sources group’ (0-5 points) and ‘a between-food group’ (0-15 points) on a categorical scale. The maximum number of points (5) was assigned to the within-protein group when there was intake of three or more different servings per day from protein sources. For other scores related to food variety, points were allocated as follows: 2 points for two different sources/d, 1 point for one source/d and 0 points for no sources. For overall food group variety, consumption of ≥ 1 serving(s) from each food group/d was allocated 3 points. Dietary adequacy was evaluated based on fruit, vegetable, grain group, fiber, protein, calcium, iron and vitamin C intake. For each nutrient, the highest tertile of intake received 5 points. A dietary moderation component was used to evaluate the intake of foods and nutrients for which consumption is likely related to chronic disease. It was comprised of 5 subgroups: saturated fat, cholesterol, total fat, empty calorie foods and sodium. For each dietary moderation item, the lowest consumption tertile was assigned 6 points. Overall dietary balance refers to the proportionality of macronutrient distribution (6 points) and composition of fatty acid (4 points) on a categorical scale. Regarding the fatty acid ratio balance in terms of polyunsaturated fatty acids/saturated fatty acids and monounsaturated fatty acids/saturated fatty acids, participants received 4 points if the ratios for both were between 1-1.5, received 2 points if the ratios for both were 0.8-1.7, and otherwise received no points. Also, the ratio of carbohydrate to protein to fat corresponding to (55-65):(10-15):(15-25) were given six points, ratios of (52-68):(9-16):(13-27) received 4 points, ratios of (50-70):(8-17):(12-30) were given 2 points, and other ratios were given zero points. The total possible DQI-I was 100 points, with higher scores indicating better dietary quality.

### Statistical analysis

Statistical analysis was done with SPSS for Windows version 16 (SPSS Inc, Chicago, IL, USA). The Kolmogorov Smirnov test was used to assess whether the variables were normally distributed. Pearson correlations were used to evaluate correlations between dietary acid load indices. Cut-off points for quartiles of dietary acid load indices were calculated and categorised based on tertile cut-off points. Significant differences in general characteristics across tertile categories of dietary acid load indices were tested using ANOVA. If there was a significant main effect, Bonferroni tests were performed to detect pairwise differences. The chi-square test was used to detect any significant differences in the distribution of participants across tertile categories of dietary acid load indices for categorical variables (i.e. prevalence of obesity, overweight, abdominal obesity and socio-economic status). To assess dietary acid load indices in relation to overweight, obesity and abdominal adiposity, we used multivariable logistic regression models. Multivariable models controlled for age (years), energy intake (kJ/d), physical activity, socioeconomic status, family history of diabetes, stroke and consumption of estrogen or dietary supplements. In the second model for overweight, obesity and abdominal adiposity risk assessment, we also adjusted for consumption of carbohydrates, fat, refined grain, and sodium.

## Results

### Participants and descriptive data

Mean and standard deviations for dietary acid load indices were -3.22 ± 14.87 mEq/d, 43.84 ± 13.62 mEq/d and 37.12 ± 15.12 mEq/d for PRAL, NEAP and DAL, respectively. The percentages of women who were overweight, obese and had abdominal obesity were 94(30.7), 38(12.4) and 126(41.2), respectively. Correlations among all three indices were quite high (PRAL and NEAP, r = 0.80, *P* < 0.001; PRAL and DAL r = 0.97, *P* < 0.001; DAL and NEAP r = 0.78, *P* < 0.001, respectively). Table 1 displays the means and standard deviations for age, physical activity and anthropometric measures, as well as the distribution of participants with respect to overweight, obesity and abdominal adiposity across quartile categories of dietary acid load indices (PRAL, NEAP and DAL). The cutoffs for dietary acid load indices were constructed as follows: tertiles for the PRAL index were ≤-7.91, > -7.91 to 3.15, and > 3.15 mEq/d;NEAP ≤37.29, 37.29< to 46.67, and > 46.67 mEq/d; and for DAL ≤31.86, 31.86< to 43.49, and > 43.49 mEq/d. Participants in the highest DAL tertile had higher weights (68.43 kg vs. 63.87 kg), heights (163.88 cm vs 161.83 cm), rates of obesity (51% vs. 35.3%) and abdominal obesity (18.6% vs. 11.8%) compared to participants in the lowest tertile.

**Table 1:** Participant characteristics across tertiles of PRAL, NEAP and DAL

**Variable** ^*^	**All participants**	**≤-7.91** **N=101**	**PRAL** **-7.91< to 3.15** **N=103**	**3.15<** **N=102**	***P***_value_^||^	**≤37.29** **N=102**	**NEAP** **37.29<to46.67** **N=101**	**46.67<** **N=103**	***P*** **value** ^||^	**≤31.86** **N=102**	**DAL** **31.86<to43.49** **N=102**	**43.49<** **N=102**	***P *** **value** ^||^
Age( year)	32.42 ± 8.31	33.01 ± 8.54	31.98 ± 8.18	32.28 ± 8.25	0.66	33.30 ± 8.54	30.90 ± 8.07	33.04 ± 8.18	0.07	32.63 ± 8.71	32.20 ± 7.93	32.44 ± 8.34	0.93
Weight( kg)	65.83 ± 12.47	66.98 ± 13.19	64.72 ± 10.46	65.81 ± 13.57	0.43	67.07 ± 12.29	65.20 ± 11.40	65.22 ± 13.63	0.47	63.87 ± 12.87	65.20 ± 10.77	68.43 ± 13.30	0.027
Height(cm)	162.88 ± 5.24	162.52 ± 5.51	163.00 ± 4.89	163.11 ± 5.34	0.69	162.85 ± 5.43	162.51 ± 5.06	163.2 ± 6 5.24	0.6	161.83 ± 5.71	162.93 ± 4.55	163.88 ± 5.24	0.02
Body mass index(kg/m^2^)	24.81 ± 4.62	25.37 ± 5.01	24.34 ± 3.77	24.73 ± 4.98	0.27	25.28 ± 4.54	24.71 ± 4.38	24.45 ± 4.94	0.42	24.40 ± 4.93	24.54 ± 3.88	25.50 ± 4.95	0.18
Waist circumference (cm)	85.44 ± 11.56	86.69 ± 13.13	84.46 ± 9.25	85.20 ± 11.99	0.37	86.55 ± 12.69	85.61 ± 10.68	84.17 ± 11.20	0.33	84.34 ± 12.79	85.11 ± 9.66	86.86 ± 11.97	0.28
Overweight^†^ (%)	94(30.7)	27(26.7)	36(35.0)	31(30.4)	0.44	29(28.4)	35(34.72)	30(29.1)	0.57	25(24.5)	34(33.3)	35(34.3)	0.24
Obesity total ^‡^ (%)	38(12.4)	16(15.8)	8(7.8)	14(13.7)	0.19	13(12.7)	12(11.9)	13(12.6)	0.98	12(11.8)	7(6.9)	19(18.6)	0.038
Grade 1	12(8.5)	10(9.9)	7(6.8)	9(8.8)		8(7.8)	10(9.9)	8(7.8)		8(7.8)	4(3.9)	14(13.7)	
Grade 2	11(3.6)	6(5.9)	1(1)	4(3.9)		5(4.9)	2(2)	4(3.9)		4(3.9)	3(2.9)	4(3.9)	
Grade 3	1(3)	0(0)	0(0)	1(1)		0(0)	0(0)	1(1)		0(0)	0(0)	0(1)	
Abdominal obesity^§^(%)	126(41.2)	42(41.6)	39(37.9)	45(44.1)	0.65	42(41.2)	43(42.6)	41(39.8)	0.92	36(35.3)	38(37.3)	52(51.0)	0.046
Physical activity (Met.h/day)	30.93 ± 3.42	30.73 ± 3.62	31.14 ± 3.26	30.92 ± 3.39	0.69	30.97 ± 3.63	30.7 ± 3.25	31.03 ± 3.39	0.86	30.9 ± 4.15	30.79 ± 2.60	31.0 ± 3.35	0.86
SES(%)	Weak	121(39.5)	43(42.6)	32(31.1)	46(45.1)	0.19	40(39.2)	34(33.7)	47(45.6)	0.23	43(42.2)	29(28.4)	49(48.0)	0.054
	Moderate	97(31.7)	29(28.7)	35(34.0)	33(32.4)		32(31.4)	31(30.7)	34(33.0)		31(30.4)	36(35.3)	30(29.4)	
	Strong	88(28.8)	29(28.7)	36(35.0)	23(22.5)		30(29.4)	36(35.6)	22(21.4)		28(27.5)	37(36.3)	23(22.5)	

MET, metabolic equivalents; PRAL, potential renal acid load; net endogenous acid production (NEAP); dietary acid load (DAL); SES, socioeconomic status ^*^ Values are mean ± SD unless indicated. ^†^ Overweight BMI ≤25<30 kg/m^2^ ^‡^Obesity BMI ≥30 kg/m^2^, obesity - grade 1 :30<BMI<34.9, obesity - grade 2 :35<BMI<39.9, obesity - grade 3 :40<BMI. ^§^Abdominal obesity: WC ≥ 88 cm. ^||^*P* values were calculated using ANOVA for continuous variables and χ^2^ for categorical variables.

### Main results

Energy-adjusted means for dietary variables (macronutrients, micronutrients and food groups) across quartile categories of dietary acid load indices are presented in Table 2. Energy intake significantly differed among PRAL and DAL tertiles. Protein, fats (cholesterol, SAFA, PUFA), refined grains, meats and sodium, were consumed in greater amounts by participants in both the high PRAL and DAL groups. Also, participants scoring in the highest tertile of dietary acid load consumed lower amounts of carbohydrates, fruits, vegetables, fiber, potassium and magnesium compared with participants in the lowest tertile of all three dietary acid load scores. Also, calcium and multivitamin supplementation did not significantly differ between tertiles of dietary acid load (results not shown).

**Table 2:** Dietary intake of women 20-50 years old across tertiles of PRAL, NEAP and DAL

**Variable** ^*^	**All** **participants**	**≤-7.91** **N=101**	**PRAL** **-7.91< to 3.15** **N=103**	**3.15<** **N=102**	***P***_value_^†^	**≤37.29** **N=102**	**NEAP** **37.29<to46.67** **N=101**	**46.67<** **N=103**	***P***_value_^†^	**≤31.86** **N=102**	**DAL** **31.86<to43.49** **N=102**	**43.49<** **N=102**	***P***_value_^†^
Energy (kcal/d)	2175.60 ± 690.70	2375.10 ± 659.39	2011.57 ± 738.11	2143.69 ± 625.86	0.001	2168.75 ± 685.44	2277.60 ± 725.06	2082.36 ± 653.28	0.12	2388.06 ± 635.98	1931.42 ± 714.80	2207.32 ± 646.86	<0.001
Protein (g)	77.31 ± 29.80	75.56 ± 15.76	74.62 ± 15.61	81.77 ± 15.53	0.002	73.21 15.43	77.16 ± 15.46	81.52 ± 15.51	0.001	75.48 ± 15.84	75.47 ± 15.94	80.99 ± 15.63	0.016
Carbohydrate (g)	330.95 ± 110.41	345.21 ± 32.42	329.31 ± 32.24	318.48 ± 31.98	<0.001	339.88 ± 33.29	329.74 ± 33.13	323.28 ± 33.15	0.002	348.68 ± 31.88	324.57 ± 32.08	319.60 ± 31.37	<0.001
Total fat(g)	65.55 ± 25.34	61.67 ± 14.45	67.14 14.39	67.79 ± 14.22	0.005	62.25 ± 14.52	65.75 ± 14.55	65.65 ± 14.50	0.96	60.09 ± 14.22	68.80 ± 14.32	67.77 ± 14.02	<0.001
Cholesterol (mg)	199.03 ± 129.42	167.32 ± 113.45	210.33 ± 112.95	219.01 ± 111.89	0.003	177.85 ± 113.91	204.26 ± 113.35	214.87 ± 113.36	0.056	157.85 ± 112.30	220.64 ± 112.90	218.59 ± 110.48	<0.001
SAFA(g)	21.56 ± 9.69	19.87 ± 6.42	22.66 ± 6.38	22.13 ± 6.35	0.006	20.99 ± 6.35	21.98 ± 6.42	21.72 ± 6.38	0.53	19.21 ± 6.35	22.94 ± 6.35	22.54 ± 6.25	<0.001
MUFA(g)	20.70 ± 8.55	19.75 ± 5.52	21.13 ± 5.47	21.22 ± 5.44	0.11	20.89 ± 5.44	20.72 ± 5.52	20.49 ± 5.47	0.86	19.20 ± 5.44	21.54 ± 5.44	21.36 ± 5.345	0.004
PUFA(g)	15.58 ± 7.41	14.56 ± 5.82	15.36 ± 5.77	16.83 ± 5.75	0.019	15.70 ± 5.85	15.19 ± 5.82	15.84 ± 5.88	0.70	14.38 ± 5.85	16.01 ± 5.85	16.34 ± 5.75	0.043
Whole grain(g)	125.56 ± 84.06	120.71 ± 81.02	121.54 ± 80.71	134.44 ± 79.91	0.39	113.89 ± 79.61	126.53 ± 79.91	136.18 ± 79.90	0.13	127.64 ± 81.02	113.76 ± 81.42	135.29 ± 79.71	0.16
Refined grain(g)	378.84 ± 145.00	347.04 ± 142.26	368.87 ± 141.65	420.41 ± 140.35	0.001	324.46 ± 137.82	392.62 ± 138.35	419.20 ± 138.30	0.0001	343.63 ± 143.17	381.49 ± 143.88	412.42 ± 140.85	0.002
Fruits(g)	373.95 ± 223.63	505.78 ± 192.96	368.03 ± 192.15	249.38 ± 190.29	<0.001	511.89 ± 187.97	361.06 ± 188.65	249.98 ± 188.50	0.0001	498.05 ± 195.74	370.02 ± 196.85	253.76 ± 192.61	<0.001
Vegetables(g)	365.49 ± 214.77	488.58 ± 186.14	339.31 ± 185.25	269.36 ± 183.63	<0.001	473.92 ± 187.47	345.57 ± 188.14	277.64 ± 187.99	<0.001	478.64 ± 189.18	345.50 ± 190.19	272.33 ± 186.16	<0.001
Dairy(g)	505.01 ± 271.90	486.25 ± 267.06	535.77 ± 265.97	492.52 ± 263.44	0.35	497.22 ± 262.74	543.00 ± 263.65	475.48 ± 263.53	0.18	474.44 ± 266.98	547.66 ± 268.39	492.93 ± 262.64	0.13
Meat & alternative(g)	96.54 ± 56.24	80.17 ± 53.71	92.41 ± 53.43	116.91 ± 52.97	<0.001	80.87 ± 53.27	93.91 ± 53.51	114.63 ± 53.43	<0.001	76.13 ± 53.88	100.74 ± 54.18	112.74 ± 52.97	<0.001
Beans & nuts(g)	43.34 ± 33.16	40.92 ± 31.32	43.63 ± 31.23	45.43 ± 30.87	0.58	43.51 ± 30.97	43.00 ± 31.124	43.49 ± 31.02	0.99	40.83 ± 31.37	42.95 ± 31.58	46.23 ± 30.87	0.45
Fiber(g)	17.53 ± 9.86	22.08 ± 6.42	16.52 ± 6.38	14.05 ± 6.35	<0.001	21.26 ± 6.55	17.06 ± 6.55	14.30 ± 6.59	<0.001	22.03 ± 6.45	16.53 ± 6.45	14.07 ± 6.35	<0.001
Phosphorus (mg)	1216.84 ± 482.62	1224.02 ± 280.31	1195.26 ± 279.05	1231.54 ± 276.46	0.61	1250.22 ± 274.85	1227.33 ± 275.89	1173.51 ± 275.70	0.12	1215.92 ± 281.20	1202.77 ± 282.72	1231.84 ± 276.66	0.75
Potassium (mg)	3223.66 ± 1417.65	3902.18 ± 725.18	3090.08 ± 722.06	2686.70 ± 715.17	<0.001	3828.73 ± 728.19	3185.07 ± 730.81	2662.32 ± 730.48	<0.001	3862.11 ± 737.67	3126.95 ± 741.61	2681.94 ± 725.67	<0.001
Calcium (mg)	1000.14 ± 410.05	1027.83 ± 283.42	1009.84 ± 282.29	962.94 ± 279.59	0.23	1032.46 ± 277.67	1026.79 ± 278.71	942.01 ± 278.54	0.036	1029.62 ± 284.63	997.64 ± 286.05	973.18 ± 279.99	0.36
Magnesium (mg)	264.99 ± 111.91	299.92 ± 62.04	255.28 ± 61.75	240.22 ± 61.14	<0.001	295.12 ± 61.34	264.70 ± 61.54	235.44 ± 61.54	<0.001	295.02 ± 62.96	261.28 ± 63.26	238.68 ± 61.95	<0.001
Sodium (mg)	5149.55 ± 2857.11	4756.80 ± 2524.65	5332.50 ± 2513.60	5353.70 ± 2489.70	<0.001	4647.03 ± 2477.59	5353.92 ± 2486.50	5446.78 ± 2485.51	0.043	4714.22 ± 2527.34	5399.97 ± 2540.66	5334.46 ± 2486.17	0.10

PRAL, potential renal acid load; NEAP, net endogenous acid production; DAL, dietary acid load; SAFA, saturated fatty acid; MUFA, monounsaturated fatty acids; PUFA, polyunsaturated fatty acids ^*^Data are mean ± SD. ^†^All P-values resulted from analysis of ANCOVA, except for energy. P-values for energy resulted from ANOVA. Significant differences among the three tertiles (*P* < 0.05; Bonferroni pairwise comparisons were done).

Multivariate linear regression analyses showed that dietary acid load (DAL), was positively associated with women’s weight and BMI (Table 3). PRAL, NEAP and DAL were strongly positively correlated with protein, meat sand meat products, and refined grains, whereas they were negatively correlated with fruits, vegetables, fiber, magnesium and potassium. Models controlled for calorie intake. With increasing dietary acid load scores (PRAL, NEAP and DAL), both of the dietary quality indices (DQI-I and MAR) significantly decreased (*P* < 0.001), whereas energy density significantly increased (*P* < 0.001) across tertile categories of dietary acid load indices. The results for dietary quality indices were similar for both crude and multivariate adjusted models (Table 4). Multivariate-adjusted odds ratios (OR) and 95% CIs for overweight, obesity and abdominal adiposity across tertile categories of dietary acid load scores are presented in Table 5. The odds of abdominal adiposity increased with tertile of DAL in the crude model (adjusted OR = 1.98, 95% CI = 1.13-3.49, *P* = 0.016). Abdominal obesity remained strongly associated with higher DAL even after controlling for confounding variables in Models 1 (adjusted OR = 2.77, 95% CI = 1.39-5.50, *P* = 0.004) and 2 (adjusted OR = 2.4, 95% CI = 1.21-4.50, *P* = 0.005). A similar relationship between the DAL score and obesity risk was found after adjustment for confounders (adjusted OR = 2.41, 95% CI = 1.01-5.74, and *P* = 0.045). Other dietary acid load indices (PRAL and NEAP) showed no significant associations with obesity, overweight or abdominal obesity.

**Table 3:** Correlations between dietary acid load and anthropometric measurements in women

**Variable**	**PRAL**				**NEAP**				**DAL**			
	**β**	***P*** ** value** ^†^	**r**	***P*** **-value** ^‡^	**β**	***P*** ** value** ^†^	**r**	***P*** **-value** ^‡^	**β**	***P*** ** value** ^†^	**r**	***P*** ** value** ^‡^
**Anthropometric indicators**												
Weight (kg)^*^	-0.02	0.56	0.03	0.55	-0.005	0.92	0.005	0.92	0.15	0.001	0.19	0.001
Height (cm)^*^	0.01	0.45	0.04	0.45	0.01	0.61	0.02	0.61	0.05	0.005	0.15	0.005
BMI ( kg/m^2^)^*^	-0.01	0.36	0.05	0.36	-0.005	0.77	0.01	0.77	0.04	0.016	0.13	0.01
WC (cm)^*^	-0.05	0.21	0.07	0.21	-0.01	0.75	0.01	0.75	0.07	0.071	0.10	0.07
**Dietary intakes**												
PRAL			1		0.04	> 0.001	0.80	> 0.001	0.95	> 0.001	0.97	> 0.001
NEAP	0.73	> 0.001	0.80	> 0.001			1		0.70	> 0.001	0.78	> 0.001
DAL	0.99	> 0.001	0.97	> 0.001	0.04	> 0.001	0.78	> 0.001			1	
Protein (g)	0.21	> 0.001	0.18	> 0.001	0.24	> 0.001	0.21	> 0.001	0.20	0.001	0.19	0.001
Carbohydrate (g)	-0.84	> 0.001	0.36	> 0.001	-0.25	0.070	0.10	0.07	-0.87	> 0.001	0.38	> 0.001
Total fat(g)	0.18	0.001	0.18	0.001	-0.09	0.13	0.08	0.13	0.20	> 0.001	0.21	> 0.001
Cholesterol(mg)	1.77	> 0.001	0.22	> 0.001	0.73	0.12	0.08	0.12	1.87	> 0.001	0.24	> 0.001
SAFA(mg)	0.06	0.005	0.15	0.006	-0.01	0.58	0.03	0.58	0.08	> 0.001	0.20	> 0.001
MUFA(mg)	0.04	0.037	0.12	0.03	-0.04	0.070	0.10	0.07	0.05	0.015	0.14	0.015
PUFA(mg)	0.06	0.004	0.16	0.004	-0.02	0.25	0.06	0.25	0.05	0.013	0.14	0.013
Whole grain(g)	0.46	0.13	0.08	0.12	0.95	0.005	0.16	0.005	0.42	0.16	0.08	0.16
Refined grain(g)	2.48	> 0.001	0.26	> 0.001	3.28	> 0.001	0.31	> 0.001	2.00	> 0.001	0.21	> 0.001
Fruits(g)	-8.35	> 0.001	0.56	> 0.001	-8.27	> 0.001	0.51	> 0.001	-7.84	> 0.001	0.54	> 0.001
Vegetables(g)	-7.20	> 0.001	0.52	> 0.001	-6.41	> 0.001	0.42	> 0.001	-6.75	> 0.001	0.49	> 0.001
Dairy(g)	0.22	0.82	0.005	0.93	-1.72	0.12	0.08	0.12	0.90	0.36	0.05	0.36
Meat & alternative(g)	1.03	> 0.001	0.27	> 0.001	0.90	> 0.001	0.22	> 0.001	1.06	> 0.001	0.29	> 0.001
Beans & nuts(g)	0.15	0.19	0.06	0.22	-0.001	0.99	0.00	0.99	0.15	0.19	0.07	0.19
Fiber(g)	-0.25	> 0.001	0.53	> 0.001	-0.20	> 0.001	0.38	> 0.001	-0.24	> 0.001	0.51	> 0.001
Phosphorus(mg)	0.97	0.36	0.03	0.52	-3.48	0.003	0.17	0.003	1.13	0.28	0.06	0.28
Potassium(mg)	38.77	> 0.001	0.46	> 0.001	-1.98	> 0.001	0.59	> 0.001	-36.61	> 0.001	0.63	> 0.001
Calcium(mg)	-1.45	0.18	0.08	0.12	-3.45	0.003	0.16	0.003	-1.08	0.31	0.05	0.31
Magnesium(mg)	-1.81	> 0.001	0.41	> 0.001	-1.98	> 0.001	0.40	> 0.001	-1.71	> 0.001	0.39	> 0.001
Sodium(mg)	18.68	0.055	0.10	0.057	18.84	0.074	0.10	0.07	13.19	0.16	0.08	0.16
DQI-I	-0.24	> 0.001	0.38	> 0.001	-1.84	> 0.001	0.26	> 0.001	-0.25	> 0.001	0.40	> 0.001
MAR	0.008	> 0.001	0.48	> 0.001	-0.009	> 0.001	0.46	> 0.001	-0.008	> 0.001	0.47	> 0.001
ED	0.006	> 0.001	0.34	> 0.001	0.007	> 0.001	0.34	> 0.001	0.005	> 0.001	0.31	> 0.001

BMI, body mass index; WC, waist circumference; PRAL, potential renal acid load; NEAP, net endogenous acid production; DAL, dietary acid load SAFA, saturated fatty acid; MUFA, monounsaturated fatty acids; PUFA, polyunsaturated fatty acids ; DQI-I, diet quality index international; MAR, mean adequacy ratio; ED, energy density. ^*^ Adjusted for total calorie intake. ^†^P-values resulted from liner regression models. ^‡^P-values resulted from Pearson correlation coefficients

**Table 4:** Dietary quality indices by tertiles of PRAL, NEAP and DAL scores in healthy women

**Variable** ^*^	**≤-7.91 N=101**	**PRAL -7.91< to 3.15** **N=103**	**3.15< N=102**	***P*** ** value** ^†^	**≤37.29 N=102**	**NEAP 37.29<to46.67** **N=101**	**46.67< N=103**	***P*** ** value** ^†^	**≤31.86** **N=102**	**DAL 31.86<to43.49** **N=102**	**43.49< N=102**	***P*** ** value** ^†^
**DQI-I**												
Crude	63.00 ± 1.14	52.75 ± 1.13	51.50 ± 1.13	> 0.001	59.21 ± 1.21	56.74 ± 1.19	51.26 ± 1.20	> 0.001	63.61 ± 1.11	51.53 ± 1.11	52.01 ± 1.11	> 0.001
Adjusted model	60.85 ± 0.89	54.29 ± 0.88	52.08 ± 0.87	> 0.001	59.23 ± 0.91	55.29 ± 0.92	52.66 ± 0.92	> 0.001	61.15 ± 0.87	54.03 ± 0.90	51.97 ± 0.87	> 0.001
**MAR**												
Crude	1.60 ± 0.03	1.23 ± 0.03	1.21 ± 0.03	> 0.001	1.48 ± 0.03	1.39 ± 0.03	1.17 ± 0.03	> 0.001	1.61 ± 0.03	1.17 ± 0.03	1.25 ± 0.03	> 0.001
Adjusted model	1.48 ± 0.01	1.32 ± 0.01	1.24 ± 0.01	> 0.001	1.49 ± 0.01	1.31 ± 0.01	1.24 ± 0.01	> 0.001	1.49 ± 0.01	1.31 ± 0.01	1.24 ± 0.01	> 0.001
**ED**												
Crude	1.02 ± 0.02	1.03 ± 0.02	1.15 ± 0.02	0.003	0.98 ± 0.02	1.07 ± 0.02	1.15 ± 0.02	> 0.001	1.04 ± 0.02	0.99 ± 0.02	1.17 ± 0.02	> 0.001
Adjusted model	0.96 ± 0.01	1.08 ± 0.01	1.16 ± 0.01	> 0.001	0.98 ± 0.01	1.06 ± 0.01	1.17 ± 0.01	> 0.001	0.99 ± 0.01	1.06 ± 0.01	1.16 ± 0.01	> 0.001

PRAL, potential renal acid load; NEAP, net endogenous acid production; DAL, dietary acid load; DQI-I, diet quality index international; MAR, mean adequacy ratio; ED, energy density. ^*^Data are mean ± SE and computed by using analysis of covariance in multivariable adjusted models. The crude model is not adjusted for any variables. Adjusted model: adjusted for age and total energy intake, physical activity, social economic status, family history of diabetes and stroke, estrogen drugs and supplements used. ^†^ P values resulted from ANCOVA test.

**Table 5:** Odds ratio and 95% confidence interval for overweight, obesity and abdominal obesity by tertiles of dietary acid load

**Variable** ^*^	**≤-7.91** **N=101**	**PRAL** **-7.91< to 3.15** **N=103**	**<3.15** **N=102**	***P***_value_^||^	**≤37.29** **N=102**	**NEAP** **37.29<to46.67** **N=101**	**<46.67** **N=103**	**P**_value_^||^	**≤31.86** **N=102**	**DAL** **31.86<to43.49** **N=102**	**<43.49** **N=102**	***P***_value_^||^
Overweight												
Crude^†^	1	1.47 (0.80-2.67)	1.19 (0.65-2.20)	0.57	1	1.33 (0.73-2.41)	1.03 (0.56-1.89)	0.91	1	1.69 (0.91-3.13)	1.69 (0.91-3.13)	0.09
Model 1^‡^	1	1.74 (0.90-3.34)	1.43 0.74-2.77)	0.28	1	1.46 (0.77-2.78)	1.10 (0.58-2.09)	0.53	1	2.28 (1.14- 4.5)	1.90 (0.98-3.67)	0.06
Model 2^§^	1	1.63 (0.87-3.08)	1.27 (0.67-2.43)	0.50	1	1.39 (0.75-2.56)	1.13 (0.60-2.10)	0.70	1	1. 90 (1.02-3.81)	1.81 (0.96-3.52)	0.08
Obesity												
Crude	1	0.44 (0.18-1.09)	0.84 (0.38-1.83)	0.65	1	0.92 (0.39-2.13)	0.98 0.43-2.25)	0.97	1	0.42 (0.15- 1.17)	1.56 (0.72-3.37)	0.20
Model 1	1	0.50 (0.15-1.63)	1.41 (0.52-3.82)	0.50	1	0.86 (0.29-2.52)	1.39 (0.49-3.94)	0.54	1	0.51 (0.14-1.86)	2.33 (0.85-6.4)	0.07
Model 2	1	0.62 (0.23-1.66)	1.30 (0.55-3.10)	0.55	1	0.84 (0.34-2.08)	1.38 (0.56-3.39)	0.48	1	0.75 (0.25- 2.22)	2.41 (1.01-5.74)	0.04
Abdominal obesity												
Crude	1	0.85 (0.48-1.50)	1.10 (0.63-1.93)	0.71	1	1.05 (0.60-1.85)	0.94 (0.54-1.65)	0.84	1	1.18 (0.66-2.09)	1.98 (1.13- 3.49)	0.016
Model 1	1	1.18 (0.60-2.32)	1.52 (0.77-2.79)	0.22	1	1.24 (0.63-2.42)	1.00 (0.52-1.93)	0.97	1	1.87 (0.92-3.8)	2.77 (1.39-5.50)	0.004
Model 2	1	1.06 (0.57-1.94)	1.30 (0.71-2.38)	0.37	1	1.03 (0.57-1.86)	1.11 (0.61-1.99)	0.72	1	1.62 (0.85- 3.08)	2.4 (1.33-4.6)	0.005

PRAL, potential renal acid load; NEAP, net endogenous acid production; DAL; Overweight BMI ≤25<30 kg/m^2^ or obesity BMI ≥30 kg/m^2^ ; Abdominally obesity: WC ≥ 88 cm. ^*^Data are Odds ratio (Confidence interval) and computed by logistic regression in multivariable adjusted models. ^†^The crude model is not adjusted for any variables. ^‡^Model 1 is adjusted for age and total energy intake, physical activity, social economic status, family history of diabetes and stroke, estrogen drugs and supplements used. ^§^Model 2 is additionally controlled for significant dietary intakes in Table 2. We controlled for the effects of carbohydrates, fat, refined grains and sodium. ^||^ *P* values are P for trend based on the Mantel-Haenszel test.

## Discussion

The findings of this cross-sectional study of Iranian women further the existing evidence regarding possible associations between dietary acid load with overweight and obesity. Our conclusions from this study are three-fold. First, our findings reveal an association between DAL and BMI, obesity and abdominal obesity. Second, in line with existing literature on this subject, we observed inconsistent relationships between overweight and specific measures of dietary acid load, noting no associations between PRAL and NEAP and weight. Third, we demonstrate, for the first time, that DAL may be used to measure dietary quality. We discovered robust associations between higher scores on ‘quality of diet’ measures DQI-I and MAR and lowered PRAL, NEAP, and DAL scores.

The positive associations between DAL with weight and BMI seen in our study are consistent with a study by Han et al^48^ However, the association we observed between DAL and waist circumference was weaker than in that study. We also found that higher levels of dietary acid load, measured with DAL, were associated with increased odds of obesity and abdominal obesity after adjustment for confounding variables. Findings regarding the other dietary acid load indices (PRAL or NEAP) were in agreement with several previous studies i.e. they revealed no significant relationship with weight, waist circumference or BMI.^55-58^ Differences in our results regarding anthropometric indices in relation to DAL (as opposed to the two other indices PRAL and NEAP) may be due to the formula used to calculate DAL, in which height and weight are directly used to compute this score.

Our results were not consistent with findings from other studies that suggested that higher PRAL score was associated with increased weight, waist circumference and/or BMI.^28,57^ For example, one previous study found an independent positive association between BMI/WC and PRAL. In that study the mean BMI of that population was higher than in our study (BMI = 27-27.3 vs. 24.8 kg/m^2^).^28^ In a study of Japanese students, higher levels of dietary acid load were significantly related to BMI/WC and pro:K (protein to potassium) ratio, but not with PRAL.^57^ Likewise, in a study by Krupp et al conducted in healthy children and adolescents, findings for PRAL were dependent on participant BMI and weight.^59^ To date, the association between dietary acid load and obesity or overweight has been examined in two cross-sectional^38,60^ and two prospective studies.^21,61^ In the cross sectional study, PRAL was not associated with the prevalence of overweight and obesity,^38^ while in other studies higher PRAL scores were significantly associated with higher rates of overweight and obesity,^21,60,61^ which was not confirmed by our study.

Differences in study results may be related to varied food patterns, different ways to measure dietary acid load, type or size of the study sample, variation in the assessment of dietary intake and to differences in which confounders were adjusted for. Moreover, in contrast to our study, prior studies were conducted in older adults (mean age = 32.4 y),^21,38,48^ except for the studies by Murakami et al^57^ and Krupp et al,^59^ which included children and adolescents. In all prior studies, older age was strongly correlated with higher dietary acid load scores. One explanation for this is that the capacity to excrete acid falls significantly with age because of decline in renal function.^21,28,38,48,62^ Another possible explanation may be that older people are inclined to consume higher acid load diets.^55,60^ Several possible mechanisms have been posited to explain the relationship between dietary acid load and obesity. First, higher dietary acid load leads to stimulation of glucocorticoid production and increased serum cortisol levels.^63^ Acid-base status affects renal magnesium losses, irrespective of magnesium intake.^64^ These processes may cause insulin resistance and subsequent overweight or obesity.^64-66^ Second, diet-induced acidosis decreases the production of adipokines (leptin and adiponectin), which can inhibit appetite suppression.^67,68^ Also, it has been demonstrated that higher dietary acid load reduces lean body mass among women and ultimately leads to higher body fat synthesis.^69^ However, these mechanisms are not clearly understood and need further investigation.

Consistent with previous studies,^48,60,70-72^ the correlations among PRAL, NEAP and DAL were quite high. This suggests that, although not identical, these measures capture similar nutrient elements. Although PRAL, NEAP and DAL are considered reasonably valid indices of dietary acid load, more research is needed to determine which is a better indicator.^22,73^ Similar to previous reserach in Iranian adults,^28^ our evaluation of dietary intake suggested that women’s diets were relatively alkaline, as indicated by median scores of -3.22 mEq/d for PRAL, 37.12 mEq/d for DAL and 43.48 mEq/d for NEAP. In contrast, in the study by Engberink et al dietary acid load was relatively high (PRAL: -1.5 mEq/d),^60^ and high in studies by Rebholz et al (PRAL:4.5, NEAP:48.0mEq/d)^61^ and Murakami et al (PRAL = 10.4mEq/d).^57^ This may have been due to the dietary pattern observed in our study that included relatively higher quantities of fruits and vegetables and lower quantities of meat, in comparison with these studies.

Recent epidemiological studies have used PRAL, NEAP and DAL scores frequently to estimate dietary acid load.^48,55^ PRAL score takes into account intestinal absorption rates nutrients that affect pH homeostasis (protein, potassium, calcium and magnesium) and the dissociation of phosphate at pH 7.4. A positive absorption rate reflected in a positive PRAL score represents acid-forming potential, whereas a negative PRAL value indicates base- (or alkaline) forming potential.^22,47^ The NEAP score is calculated by taking the dietary intake ratio of protein to potassium consumed.^51^ The DAL score combines PRAL and body surface area.^48^ For all three dietary acid load scores, increasing values measure greater consumption of acid-inducing foods.

We observed an independent negative? association between all three dietary acid load scores and DQI-I and MAR. To our knowledge, this is the first study to assess how dietary acid load and dietary quality indices are associated. Our results were similar to findings from many studies that showed that higher intakes of meats, refined grains, fish, egg and fat groups were associated with higher PRAL, NEAP and DAL, and that the higher intakes of fruits and vegetables were related to lower scores.^56,57,61^ Conversely, more than half of the points scored on DQI-I correspond to the consumption of fruits, vegetables, calcium and low intake of simple carbohydrates and fats (total fat, cholesterol and SAFA). These nutrients were significantly associated with dietary acid load scores (PRAL, NEAP and DAL) in the present study. Also, the MAR score was negatively associated with PRAL, NEAP and DAL. The MAR score typically includes two nutrient categories influence dietary acid load scores. The first includes potassium, calcium and magnesium, which are responsible for lower levels of dietary acid load. The second group of nutrients is positively associated with dietary acid load and includes protein and phosphorus. Although other nutrients included in the MAR score are not directly calculated in dietary acid load, they can indirectly contribute to the dietary acid load estimate because they represent protein, fruits and vegetable food sources. It appears that our study population consumed larger quantities of dietary sources containing acid-reducing factors (fruits and vegetables) than food sources with protein and phosphorus. ED was the third dietary quality index that we investigated in this study. In contrast with DQI-I and MAR, ED had a positive relationship with dietary acid load scores. ED represents of the amount of calories in one gram of total daily food intake.^54^ In a previous cross-sectional study, fat and protein intake appeared to be positively associated with energy intake when energy density was replaced by its nutrient correlates.^74^ At the same time, fiber and water intakes were negatively associated with energy intake. Therefore, our study revealed that greater dietary acid load due to higher consumption of protein leads to increases in ED score. Several studies have reported that healthy dietary patterns, such as the Mediterranean diet and Dietary Approaches to Stop Hypertension (DASH) that are high in fruits and vegetables increase diet quality.^75,76^

## Conclusion

In conclusion, we assessed diet-induced acidosis using several methods: PRAL, NEAP and DAL scores. In our study, the correlations between PRAL, NEAP and DAL scores were high (Pearson correlation coefficient > 0.78, *P* < 0.001), which is consistent with previous studies (r = 0.95, *P* < 0.001).^62,77^ A main limitation of our study is its cross-sectional design, which does not allow for analysis of cause and effect. Second, categorization of dietary acid load scores into tertiles could lead to data misclassification. Also, the semi-quantitative FFQ could be prone to recall bias suggesting that misclassification is another limitation.

We found that higher DAL was associated with higher risk of obesity and abdominal obesity in Iranian women after adjusting for possible confounding factors. Also, independent positive associations between PRAL, NEAP, DAL (more acidic dietary acid–base loads) and DQI-I and MAR were observed. Furthermore, diet-induced acidosis was associated with increased ED scores. Our findings show that these dietary acid load indices can be considered indicators of dietary quality. To confirm these results, the associations between dietary acid load and dietary quality indices should be tested in future prospective studies and clinical trials. Since this study was limited to healthy women, more research is needed to generalize these results to the whole population, These results can help guide clinical and policy guidelines for the adoption of appropriate dietary patterns to prevent and control weight loss and obesity.

## Acknowledgments

We thank the participants who took part in this study. We would like to express our gratitude to Dr. Mohammad Shariati, heath assistant, Zahra Beygom Aghamiri, secretary of the Health Research Council of Tehran University of Medical Sciences, and all staff members at the participating health centers. The reporting of this research is compliant with strengthening the Reporting of Observational Studies in Epidemiology (STROBE) guidelines.

## Competing Interest

None of the authors have any conflicts of interest. The study has been conducted in accordance with the ethical standards of the Helsinki Declaration.

## Ethical approval

The study carried out in accordance with the ethical standards as outlined in the Helsinki Declaration. All participants provided informed consent. Ethics Committee of Tehran University of Medical Science approved the study protocol (Grant number: 95-02-161-32458) and the protocol was carried out in accordance with the ethical standards outlined in the Helsinki Declaration.

## Funding

The protocol of the study was approved by the Ethics Committee of Tehran University of Medical Science (TUMS) (Grant number: 9411323011)

## References

[1] James WP (2008). The epidemiology of obesity: the size of the problem. J Intern Med.

[2] Nguyen DM, El-Serag HB (2010). The epidemiology of obesity. Gastroenterol Clin North Am.

[3] Tanner RM, Brown TM, Muntner P (2012). Epidemiology of obesity, the metabolic syndrome, and chronic kidney disease. Curr Hypertens Rep.

[4] Azizi F, Azadbakht L, Mirmiran P (2005). Trends in overweight, obesity, and central obesity among adults residing in district 13 of Tehran: Tehran Lipid and Glucose Study. Research in Medicine.

[5] Hales CM, Fryar CD, Carroll MD, Freedman DS, Ogden CL (2018). Trends in obesity and severe obesity prevalence in US youth and adults by sex and age, 2007-2008 to 2015-2016. JAMA.

[6] Salimi Y, Taghdir M, Sepandi M, Karimi Zarchi AA (2019). The prevalence of overweight and obesity among Iranian military personnel: a systematic review and meta-analysis. BMC Public Health.

[7] Vaisi-Raygani A, Mohammadi M, Jalali R, Ghobadi A, Salari N (2019). The prevalence of obesity in older adults in Iran: a systematic review and meta-analysis. BMC Geriatr.

[8] Shoelson SE, Herrero L, Naaz A (2007). Obesity, inflammation, and insulin resistance. Gastroenterology.

[9] Swinburn BA, Sacks G, Hall KD, McPherson K, Finegood DT, Moodie ML (2011). The global obesity pandemic: shaped by global drivers and local environments. Lancet.

[10] Saraf Bank S, Ghanjali N, Seyyed Ghalaeh R, Azadbakht L (2013). Association between dairy and calcium intake and general and central obesity among female students. J Educ Health Promot.

[11] Hajihashemi P, Azadbakht L, Hashemipor M, Kelishadi R, Esmaillzadeh A (2014). Whole-grain intake favorably affects markers of systemic inflammation in obese children: a randomized controlled crossover clinical trial. Mol Nutr Food Res.

[12] Mytton OT, Nnoaham K, Eyles H, Scarborough P, Ni Mhurchu C (2014). Systematic review and meta-analysis of the effect of increased vegetable and fruit consumption on body weight and energy intake. BMC Public Health.

[13] Jacobs DR Jr, Gross MD, Tapsell LC (2009). Food synergy: an operational concept for understanding nutrition. Am J Clin Nutr.

[14] Salas-Salvadó J, Martinez-González M, Bulló M, Ros E (2011). The role of diet in the prevention of type 2 diabetes. Nutr Metab Cardiovasc Dis.

[15] Tucker KL (2010). Dietary patterns, approaches, and multicultural perspective. Appl Physiol Nutr Metab.

[16] Williams RS, Heilbronn LK, Chen DL, Coster AC, Greenfield JR, Samocha-Bonet D (2016). Dietary acid load, metabolic acidosis and insulin resistance - lessons from cross-sectional and overfeeding studies in humans. Clin Nutr.

[17] Della Guardia L, Roggi C, Cena H (2016). Diet-induced acidosis and alkali supplementation. Int J Food Sci Nutr.

[18] Berkemeyer S (2009). Acid-base balance and weight gain: are there crucial links via protein and organic acids in understanding obesity?. Med Hypotheses.

[19] Remer T (2000). Influence of diet on acid-base balance. Semin Dial.

[20] Sebastian A, Frassetto LA, Sellmeyer DE, Merriam RL, Morris RC Jr (2002). Estimation of the net acid load of the diet of ancestral preagricultural Homo sapiens and their hominid ancestors. Am J Clin Nutr.

[21] Fagherazzi G, Vilier A, Bonnet F, Lajous M, Balkau B, Boutron-Rualt MC (2014). Dietary acid load and risk of type 2 diabetes: the E3N-EPIC cohort study. Diabetologia.

[22] Remer T, Dimitriou T, Manz F (2003). Dietary potential renal acid load and renal net acid excretion in healthy, free-living children and adolescents. Am J Clin Nutr.

[23] Remer T, Manz F (1995). Potential renal acid load of foods and its influence on urine pH. J Am Diet Assoc.

[24] Banerjee T, Crews DC, Wesson DE, Tilea A, Saran R, Rios Burrows N (2014). Dietary acid load and chronic kidney disease among adults in the United States. BMC Nephrol.

[25] Hayata H, Miyazaki H, Niisato N, Yokoyama N, Marunaka Y (2014). Lowered extracellular pH is involved in the pathogenesis of skeletal muscle insulin resistance. Biochem Biophys Res Commun.

[26] Robey IF (2012). Examining the relationship between diet-induced acidosis and cancer. Nutr Metab (Lond).

[27] Williams RS, Kozan P, Samocha-Bonet D (2016). The role of dietary acid load and mild metabolic acidosis in insulin resistance in humans. Biochimie.

[28] Bahadoran Z, Mirmiran P, Khosravi H, Azizi F (2015). Associations between dietary acid-base load and cardiometabolic risk factors in adults: the Tehran Lipid and Glucose Study. Endocrinol Metab (Seoul).

[29] Vezzoli G, Dogliotti E, Terranegra A, Arcidiacono T, Macrina L, Tavecchia M (2015). Dietary style and acid load in an Italian population of calcium kidney stone formers. Nutr Metab Cardiovasc Dis.

[30] Zhang L, Curhan GC, Forman JP (2009). Diet-dependent net acid load and risk of incident hypertension in United States women. Hypertension.

[31] Han E, Kim G, Hong N, Lee YH, Kim DW, Shin HJ (2016). Association between dietary acid load and the risk of cardiovascular disease: nationwide surveys (KNHANES 2008-2011). Cardiovasc Diabetol.

[32] Kim S, Haines PS, Siega-Riz AM, Popkin BM (2003). The Diet Quality Index-International (DQI-I) provides an effective tool for cross-national comparison of diet quality as illustrated by China and the United States. J Nutr.

[33] Azadbakht L, Esmaillzadeh A (2009). Diet variety: a measure of nutritional adequacy and health. J Qazvin Univ Med Sci.

[34] Ashouri M, Jahangosha F, Hassanzadeh A, Esmaillzadeh A (2011). Dietary energy density in relation to obesity. Health Syst Res.

[35] Murakami K, Livingstone MB (2016). Energy density of meals and snacks in the British diet in relation to overall diet quality, BMI and waist circumference: findings from the National Diet and Nutrition Survey. Br J Nutr.

[36] Sundararajan K, Campbell MK, Choi YH, Sarma S (2014). The relationship between diet quality and adult obesity: evidence from Canada. J Am Coll Nutr.

[37] Aghasi M, Motlagh AD, Eshraghian M, Mansouri P (2013). Food insecurity and some scio-economic factors affecting women suffering from acne. Nationalpark-Forschung in der Schweiz.

[38] Haghighatdoost F, Mortazavi Najafabadi M, Bellissimo N, Azadbakht L (2015). Association of dietary acid load with cardiovascular disease risk factors in patients with diabetic nephropathy. Nutrition.

[39] Jafari-Adli S, Jouyandeh Z, Qorbani M, Soroush A, Larijani B, Hasani-Ranjbar S (2014). Prevalence of obesity and overweight in adults and children in Iran; a systematic review. J Diabetes Metab Disord.

[40] Oh SW, Shin SA, Yun YH, Yoo T, Huh BY (2004). Cut-off point of BMI and obesity-related comorbidities and mortality in middle-aged Koreans. Obes Res.

[41] Okosun IS, Rotimi CN, Forrester TE, Fraser H, Osotimehin B, Muna WF (2000). Predictive value of abdominal obesity cut-off points for hypertension in blacks from west African and Caribbean island nations. Int J Obes Relat Metab Disord.

[42] Willett WC, Sampson L, Stampfer MJ, Rosner B, Bain C, Witschi J (1985). Reproducibility and validity of a semiquantitative food frequency questionnaire. Am J Epidemiol.

[43] Esmaillzadeh A, Kimiagar M, Mehrabi Y, Azadbakht L, Hu FB, Willett WC (2007). Dietary patterns, insulin resistance, and prevalence of the metabolic syndrome in women. Am J Clin Nutr.

[44] Esmaillzadeh A, Azadbakht L (2008). Food intake patterns may explain the high prevalence of cardiovascular risk factors among Iranian women. J Nutr.

[45] Ghaffarpour M, Houshiar-Rad A, Kianfar H (1999). The Manual for Household Measures, Cooking Yields Factors and Edible Portion of Foods. Tehran: Nashre Olume Keshavarzy.

[46] Ainsworth BE, Haskell WL, Whitt MC, Irwin ML, Swartz AM, Strath SJ (2000). Compendium of physical activities: an update of activity codes and MET intensities. Med Sci Sports Exerc.

[47] Remer T, Manz F (1994). Estimation of the renal net acid excretion by adults consuming diets containing variable amounts of protein. Am J Clin Nutr.

[48] Han E, Kim G, Hong N, Lee YH, Kim DW, Shin HJ (2016). Association between dietary acid load and the risk of cardiovascular disease: nationwide surveys (KNHANES 2008-2011). Cardiovasc Diabetol.

[49] Du Bois D, Du Bois EF (1989). A formula to estimate the approximate surface area if height and weight be known 1916. Nutrition.

[50] Verbraecken J, Van de Heyning P, De Backer W, Van Gaal L (2006). Body surface area in normal-weight, overweight, and obese adults A comparison study. Metabolism.

[51] Frassetto LA, Todd KM, Morris RC, Jr Jr, Sebastian A (1998). Estimation of net endogenous noncarbonic acid production in humans from diet potassium and protein contents. Am J Clin Nutr.

[52] Hatløy A, Torheim LE, Oshaug A (1998). Food variety--a good indicator of nutritional adequacy of the diet? a case study from an urban area in Mali, West Africa. Eur J Clin Nutr.

[53] Azadbakht L, Haghighatdoost F, Esmaillzadeh A (2012). Dietary energy density is inversely associated with the diet quality indices among Iranian young adults. J Nutr Sci Vitaminol (Tokyo).

[54] Azadbakht L, Esmaillzadeh A (2012). Dietary energy density is favorably associated with dietary diversity score among female university students in Isfahan. Nutrition.

[55] Luis D, Huang X, Riserus U, Sjögren P, Lindholm B, Arnlöv J (2015). Estimated dietary acid load is not associated with blood pressure or hypertension incidence in men who are approximately 70 years old. J Nutr.

[56] Iwase H, Tanaka M, Kobayashi Y, Wada S, Kuwahata M, Kido Y (2015). Lower vegetable protein intake and higher dietary acid load associated with lower carbohydrate intake are risk factors for metabolic syndrome in patients with type 2 diabetes: post-hoc analysis of a cross-sectional study. J Diabetes Investig.

[57] Murakami K, Sasaki S, Takahashi Y, Uenishi K (2008). Association between dietary acid-base load and cardiometabolic risk factors in young Japanese women. Br J Nutr.

[58] Khalili Moghadam S, Bahadoran Z, Mirmiran P, Tohidi M, Azizi F (2016). Association between dietary acid load and insulin resistance: Tehran Lipid and Glucose Study. Prev Nutr Food Sci.

[59] Krupp D, Shi L, Maser-Gluth C, Pietzarka M, Remer T (2013). 11β Hydroxysteroid dehydrogenase type 2 and dietary acid load are independently associated with blood pressure in healthy children and adolescents. Am J Clin Nutr.

[60] Engberink MF, Bakker SJ, Brink EJ, van Baak MA, van Rooij FJ, Hofman A (2012). Dietary acid load and risk of hypertension: the Rotterdam Study. Am J Clin Nutr.

[61] Rebholz CM, Coresh J, Grams ME, Steffen LM, Anderson CA, Appel LJ (2015). Dietary acid load and incident chronic kidney disease: results from the ARIC study. Am J Nephrol.

[62] Akter S, Eguchi M, Kurotani K, Kochi T, Pham NM, Ito R (2015). High dietary acid load is associated with increased prevalence of hypertension: the Furukawa Nutrition and Health Study. Nutrition.

[63] Wahl P, Zinner C, Achtzehn S, Bloch W, Mester J (2010). Effect of high- and low-intensity exercise and metabolic acidosis on levels of GH, IGF-I, IGFBP-3 and cortisol. Growth Horm IGF Res.

[64] Rylander R, Remer T, Berkemeyer S, Vormann J (2006). Acid-base status affects renal magnesium losses in healthy, elderly persons. J Nutr.

[65] Paolisso G, Barbagallo M (1997). Hypertension, diabetes mellitus, and insulin resistance: the role of intracellular magnesium. Am J Hypertens.

[66] Hotamisligil GS, Shargill NS, Spiegelman BM (1993). Adipose expression of tumor necrosis factor-alpha: direct role in obesity-linked insulin resistance. Science.

[67] Robey IF (2012). Examining the relationship between diet-induced acidosis and cancer. Nutr Metab (Lond).

[68] Arner P (2005). Resistin: yet another adipokine tells us that men are not mice. Diabetologia.

[69] Faure AM, Fischer K, Dawson-Hughes B, Egli A, Bischoff-Ferrari HA (2017). Gender-specific association between dietary acid load and total lean body mass and its dependency on protein intake in seniors. Osteoporos Int.

[70] Remer T (2004). Estimates of renal net acid excretion and bone health. Am J Clin Nutr.

[71] Macdonald HM, New SA, Fraser WD, Campbell MK, Reid DM (2005). Low dietary potassium intakes and high dietary estimates of net endogenous acid production are associated with low bone mineral density in premenopausal women and increased markers of bone resorption in postmenopausal women. Am J Clin Nutr.

[72] Welch AA, Bingham SA, Reeve J, Khaw KT (2007). More acidic dietary acid-base load is associated with reduced calcaneal broadband ultrasound attenuation in women but not in men: results from the EPIC-Norfolk cohort study. Am J Clin Nutr.

[73] Banerjee T, Crews DC, Wesson DE, Tilea AM, Saran R, Ríos-Burrows N (2015). igh dietary acid load predicts ESRD among adults with CKD. J Am Soc Nephrol.

[74] Stookey JD (2001). Energy density, energy intake and weight status in a large free-living sample of Chinese adults: exploring the underlying roles of fat, protein, carbohydrate, fiber and water intakes. Eur J Clin Nutr.

[75] Veissi M, Anari R, Amani R, Shahbazian H, Latifi SM (2016). Mediterranean diet and metabolic syndrome prevalence in type 2 diabetes patients in Ahvaz, southwest of Iran. Diabetes Metab Syndr.

[76] Schwingshackl L, Bogensberger B, Hoffmann G (2018). Diet quality as assessed by the healthy eating index, alternate healthy eating index, dietary approaches to stop hypertension score, and health outcomes: an updated systematic review and meta-analysis of cohort studies. J Acad Nutr Diet.

[77] Luis D, Huang X, Riserus U, Sjögren P, Lindholm B, Arnlöv J (2015). Estimated dietary acid load is not associated with blood pressure or hypertension incidence in men who are approximately 70 years old. J Nutr.

